# Home, Identity, and Aging: The Immigrant Experience

**DOI:** 10.1093/geroni/igaf122.1242

**Published:** 2025-12-31

**Authors:** Rong Fu, Marco Psyllos

**Affiliations:** Siena University, Loudonville, New York, United States; Bard College, Loudonville, New York, United States

## Abstract

The need to facilitate aging in place for the growing aging population has gained increasing global attention in both policy and practice. However, there is a significant gap in research regarding older immigrants’ perceptions of home and identity, which are essential for understanding both individual- and community-level influences on aging in place. This study systematically synthesizes qualitative evidence on older immigrants’ conceptualizations of home and their positive and negative experiences of aging in a foreign country. A systematic review of literature was conducted by searching JSTOR, Medline, PsycINFO, and PubMed. Eligible studies were appraised using the JBI Critical Appraisal Checklist for Qualitative Research, resulting in the inclusion of 21 studies. Findings indicate that older immigrants have diverse experiences regarding their sense of home and belonging within their current communities. Home is conceptualized in multiple ways, including as a physical dwelling, a sense of belonging and security, the presence of family, and the preservation of cultural identity. Negative experiences were associated with factors such as insecurity, inadequate support, and social isolation, whereas positive experiences were shaped by the presence of significant others and enhanced access to social welfare. This review highlights the importance of fostering supportive and inclusive communities to better align with the needs of older immigrants aging in place.

